# Artificial Intelligence for Anesthesia: What the Practicing Clinician Needs to Know

**DOI:** 10.1097/ALN.0000000000002384

**Published:** 2018-10

**Authors:** Michael R. Mathis, Sachin Kheterpal, Kayvan Najarian

**Affiliations:** 1 Department of Anesthesiology, University of Michigan Health System; 2 Department of Computational Medicine and Bioinformatics, and the Department of Emergency Medicine, University of Michigan Health System

## Editorial

Machine learning, the quintessential tool currently driving forward the development of artificial intelligence, was discovered and developed decades ago. Nevertheless, only recently has machine learning seen an exponential increase in growth, sophistication, and influence. Recent success stories outside healthcare are numerous, including: in 2014 Facebook unveiled DeepFace, a machine learning technology capable of identifying faces with 97.25% accuracy (compared to human accuracy of 97.53%).^1^ In 2016 Google adopted a deep learning approach to language translation, using an algorithm which is fed massive amounts of data to effectively train itself to recognize patterns in speech, with a reduction in translation errors by 87%.^2^

Machine learning techniques like these may be coming soon to an operating room near you: in this issue, we explore three examples of machine learning applied to our field. These include works by Lee *et al.*, using machine learning techniques to predict postoperative mortality from electronic health record data,^3^ and works by Kendale *et al.*, and Hatib *et al.*, predicting hypotension through machine learning algorithms leveraging data available during induction of anesthesia^4^ and high-fidelity arterial line waveforms,^5^ respectively. Previously, in the March 2018 issue of *Anesthesiology*, Lee *et al*. used machine learning to predict bispectral index values produced by target-controlled infusions of propofol and remifentanil.^6^ An accompanying editorial provided a valuable summary of the history of artificial intelligence and an introduction to machine learning, the component of artificial intelligence that allows computers to make what humans describe as intelligent choices and predictions.^7^ Although disagreement exists whether artificial intelligence, as driven by machine learning algorithms, portends an optimistic or ominous future, it is indisputable that machine learning paradigms have gained widespread traction in every industry.

Within the works featured in this issue, a rich underlying digital health dataset enabled the authors to leverage properties of machine learning to study old problems in new ways. These machine learning properties include an ability to capture numerous variables, better known as machine learning model features, which would otherwise elude human abilities to perceive or simultaneously consider (as is the case for the 2.6 million arterial waveform combinatorial features described by Hatib *et al*.). These also include the ability of machine learning to model complex relationships between model features which otherwise eclipse human understanding (as is the case for the deep neural network model described by Lee *et al.*).

Although some “transparent” machine learning methods provide insight into associations discovered, machine learning predictive models by nature do not require human comprehension in order to work. An ensuing challenge for scientific progress over the next decade will be to create and enforce standards for evaluating these methods, so as not to supersede the ability of authors to explain, or readers to understand. Concurrent with the rise of Big Data has been a rise in the inconsistency and uncertainty of applying machine learning concepts to datasets. If not kept in check, spurious conclusions drawn from methodologically unsound studies threaten the credibility of this science. Answering this call to action, and importantly recognized by all three featured articles, are a set of multidisciplinary guidelines for developing and reporting machine learning predictive models in biomedical research – well worth the read.^8^

**Figure 1:**
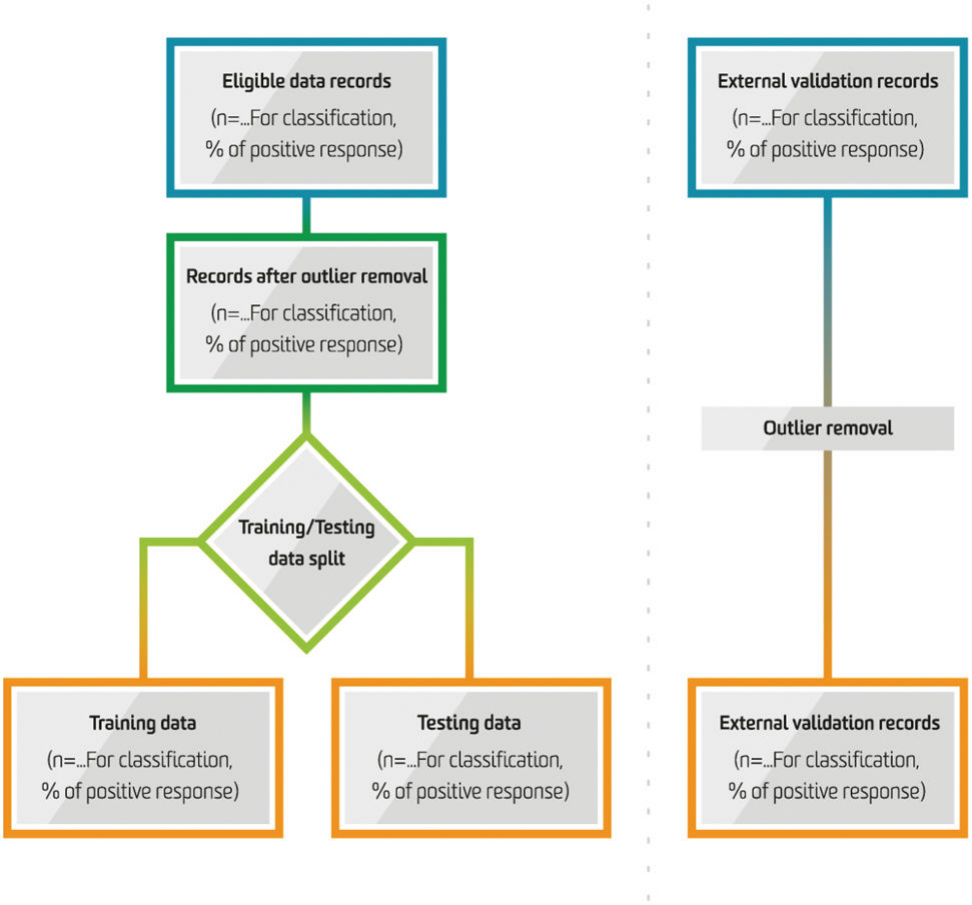
Information Flow in the Predictive Modelling Process for Machine Learning. Adapted from Luo W, Phung D, Tran T, Luo W, Phung D, Tran T, Gupta S, Rana S, Karmakar C, Shilton A, Yearwood J, Dimitrova N, Ho TB, Venkatesh S, Berk M. Guidelines for Developing and Reporting Machine Learning Predictive Models in Biomedical Research: A Multidisciplinary View. *Journal of medical Internet research.* 2016;18(12):e323.

Beyond a dire need for reporting standards in machine learning predictive models, it is of equal burden for practitioners to have a basic literacy of machine learning concepts in order to appraise machine learning-based investigations, much in the same way current biomedical literature demands a basic literacy of classical statistics and study design. These machine learning concepts include the use of training, testing, and validation datasets – used respectively to develop, assess internal performance, and externally validate machine learning algorithms (Figure 1). Additionally, just as clinicians are familiar with conventional statistical analyses such as logistic regression (which consequently, happens to be one simple type of algorithm supported by machine learning), it may behoove the perioperative clinician to be familiar with other machine learning techniques, including naïve Bayes, support vector machines, and random forests – to name a few; others are highlighted by Kendale *et al.* in this issue.

As demonstrated by the studies in this issue, the principal advantage of machine learning is the boost in performance it achieves when attempting to predict an observed outcome for which the range of explanatory features is large, or the depth of interactions between features is overwhelmingly complex. To predict hypotension, Hatib *et al.* brilliantly tap into vast arrays of data within the arterial line waveform, extending far beyond simple characteristics such as heart rate and blood pressure (and furthermore, far beyond “complex” characteristics such as pulse pressure variation, systolic pressure rise [dP/dt], and waveform area). When posed with an analytic task in which potential predictive features are in the thousands or millions or of nuanced complexity, the flexibility of machine learning techniques to accommodate inputs simply outmatch any traditional analytic method. In biomedical literature, other fields leveraging machine learning to tackle complex tasks include image processing (e.g. computer vision) of radiographic^9^ or whole-slide pathology^10^ images, as well as text analysis (natural language processing) of clinical notes.^11,12^

In contrast, for predictive analytic tasks in which features remain countable, or relationships explainable, machine learning may still prove useful, but will likely be of more modest benefit. In the work by Kendale *et al.*, an ensemble of machine learning methods indeed outperformed a classic logistic regression approach for predicting hypotension, but the overall performance of the machine learning model remained far from perfect. In the case of the best-performing algorithm (gradient boosting machines), Kendale *et al.* demonstrate a relatively small improvement compared to a classic logistic regression approach. Similarly, whereas Lee *et al.* successfully demonstrate a deep learning approach to predicting postoperative mortality from intraoperative data, the authors fail to demonstrate improvement compared to logistic regression, a recurring issue in studies promoting the use of deep learning.

As with all methodological approaches, machine learning is not without drawbacks. The most hotly contested is the difficulty of understanding mechanisms driving the prediction models presented. Herein lies the “black magic” of machine learning: although the predictive performance of a machine learning algorithm can be precisely quantified – and sometimes, this performance is staggering – the question of how to interpret and act upon the information generated remains wholly unanswered. In cases where mechanisms are of limited concern, or penalties for incorrect predictions low – such as facial recognition in family photos – machine learning techniques deftly succeed in their purpose. Conversely, in cases where mechanisms are critical, and penalties for error are high – as is often the case in healthcare, and particularly in anesthesiology – a machine learning approach falling anywhere short of nearly perfect remains unviable. Hatib *et al.* importantly note that although prediction of hypotension can be established with high fidelity, it remains entirely unclear as to how a clinician should respond to such an alert. This issue is even more critical, considering the generalizability and reproducibility concerns of such models. In many studies leveraging machine learning, insufficient testing and validation of complex models – particularly those using deep learning – can lead to overfitting of even the largest of datasets.

Despite such limitations, the work in this issue takes courageous shifts in methodologic approaches, and unmistakably establishes that machine learning applications to anesthesiology are not just a fad. The authors should be commended as exemplars for assertively applying new scientific paradigms to our field. How such machine learning techniques are harnessed in order to improve anesthesia, and more broadly advance health sciences, remains a challenge for decades to come.
